# A systematic review and meta-analysis of socio-cognitive impairments in multiple sclerosis

**DOI:** 10.1038/s41598-024-53750-5

**Published:** 2024-03-26

**Authors:** Mandy Roheger, Lydia Grothe, Laura Hasselberg, Matthias Grothe, Marcus Meinzer

**Affiliations:** 1 Department of Neurology, University Medicine Greifswald, Walther Rathenau Str. 49, 17489 Greifswald, Germany; 2 Ambulatory Assessment in Psychology, Department of Psychology, Carl Von Ossietzky University Oldenburg, Oldenburg, Germany

**Keywords:** Multiple sclerosis, MS, Social cognition, Theory of mind, Systematic review, Magnetic resonance imaging, Functional magnetic resonance imaging, Cognitive neuroscience, Neuroscience, Social behaviour, Psychology

## Abstract

Socio-cognitive impairment is frequent in multiple sclerosis (MS). However, little is known about the relationship between other potentially relevant clinical symptoms (i.e., cognition, depression, fatigue) and the degree of socio-cognitive impairment, and neural mechanisms underlying socio-cognitive deficits in MS. Therefore, we meta-analytically quantified socio-cognitive impairment in MS. A systematic literature search in MEDLINE Ovid, Web of Science Core Collection, CENTRAL, and PsycInfo was conducted until December 2022. Studies investigating affective or cognitive theory of mind (a/cToM), visual perspective taking (VPT) and social decision making (SDM) in MS patients relative to healthy controls were included. Risk-of-bias (RoB) was assessed using the CLARITY group “Tool for Assessing RoB in Cohort Studies”. Mediation analysis investigated the contribution of clinical symptoms to socio-cognitive impairment. In total, n** = **8534 studies were screened, 58 were included in the systematic review, 27 in the meta-analyses. Most studies were rated with a moderate RoB. Meta-analyses confirmed impairment of both aToM and cToM in MS patients, with larger effect sizes for aToM. Mediation analysis demonstrated that higher levels of fatigue selectively predicted the degree of cToM impairment. There was insufficient data available to quantify impairment in other socio-cognitive domains. Fourteen structural and functional imaging studies were identified and characterized by substantial heterogeneity. Summarized, this study confirmed substantial socio-cognitive impairment in MS and highlights the potential exacerbating role of comorbid clinical symptoms. We identify several evidence gaps that need to be addressed in future large-scale studies using comprehensive and coordinated assessments of socio-cognitive parameters, potential mediators, and neural correlates.

**Trial registration:** The pre-registered review protocol can be assessed at www.crd.york.ac.uk/PROSPERO/ (ID: CRD42020206225).

## Introduction

Multiple sclerosis (MS) is a chronic and progressive neurodegenerative disease leading to heterogenous neurological deficits^1^, including up to 70% of patients suffering from cognitive impairment^2^, fatigue^3^ or depression^4^. Besides these well-documented symptoms, impairment of social cognition (SC), an umbrella term describing how people process, store, and apply information relevant to social interactions^5^, have also been reported in MS^6,7^. For example, MS-patients may have problems understanding the emotions of others (affective Theory of Mind , aToM), or their cognitive states, beliefs, thoughts, or intentions (cognitive Theory of Mind, cToM)^8^. Several, recent meta-analyses have demonstrated moderate effect sizes for impairment of both aToM and cToM in patients with MS compared to healthy controls^9–11^. In the clinical presentation of MS-patients, there is a strong interplay between cognition, fatigue, and depression, which often complicates diagnostic evaluation and initiating adequate treatment^12^. However, the potential impact of these symptoms on socio-cognitive impairment has not yet been addressed. Moreover, while there is an extensive literature on the functional and structural brain correlates underlying impaired cognition^8^, fatigue^13^, and depression^14^ in MS, only a few studies have used brain imaging methods, such as functional and structural magnetic resonance imaging (MRI), to investigate the neural underpinnings of socio-cognitive deficits in MS.

To address these open issues, we initially performed a systematic review of all studies assessing SC in patients with MS across three broad socio-cognitive domains (i.e., social perception, social understanding and social decision making^15^), investigated methodological biases and conducted a meta-analysis to quantify the degree of socio-cognitive impairment in MS relative to healthy controls. In a second step, we conducted a meta-regression analyses to identify potential mediators of socio-cognitive impairment in MS. Finally, we also provide an overview of studies that also investigated the functional and structural correlates of SC impairment in MS using MRI based measures.

## Results

### Search results

**Figure 1:**
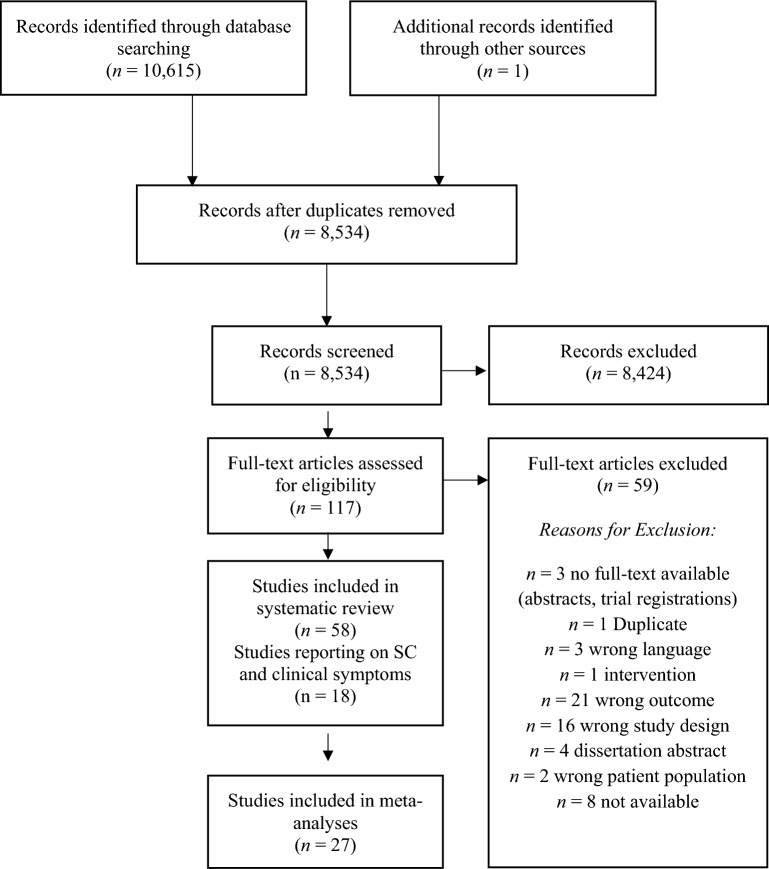
PRISMA Diagram of the study selection process.

The search strategy yielded 10,615 articles (including the results of the update search). After deduplication, 8,534 unique articles had their titles and abstracts assessed for eligibility. From these articles, 8,424 articles were ineligible. 117 full-texts were assessed further and 58 studies included in the systematic review (Table 1), 14 of which also provided information on imaging data and the neural correlates of SC (Table 2). 27 studies were included in pairwise meta-analyses, 18 studies providing information on both social cognition and clinical outcomes in the mediation analysis. The PRISMA flow-diagram^16^ in Fig. 1 illustrates the study selection process.

**Table 1:**
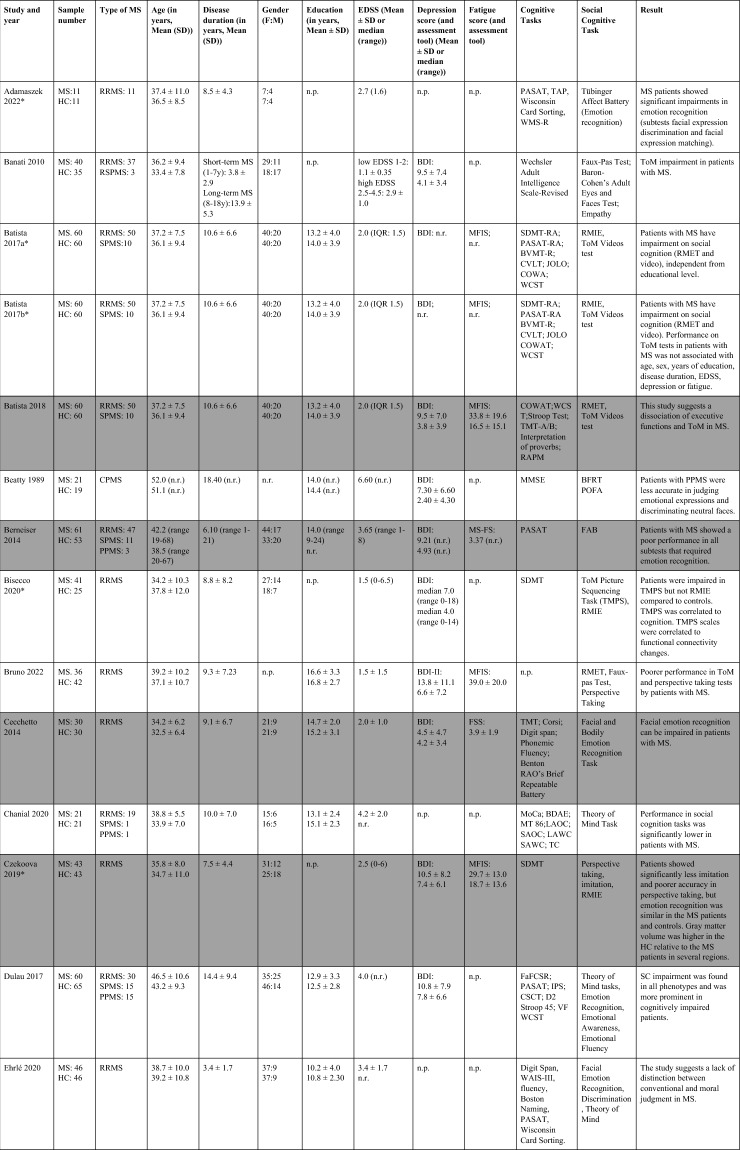

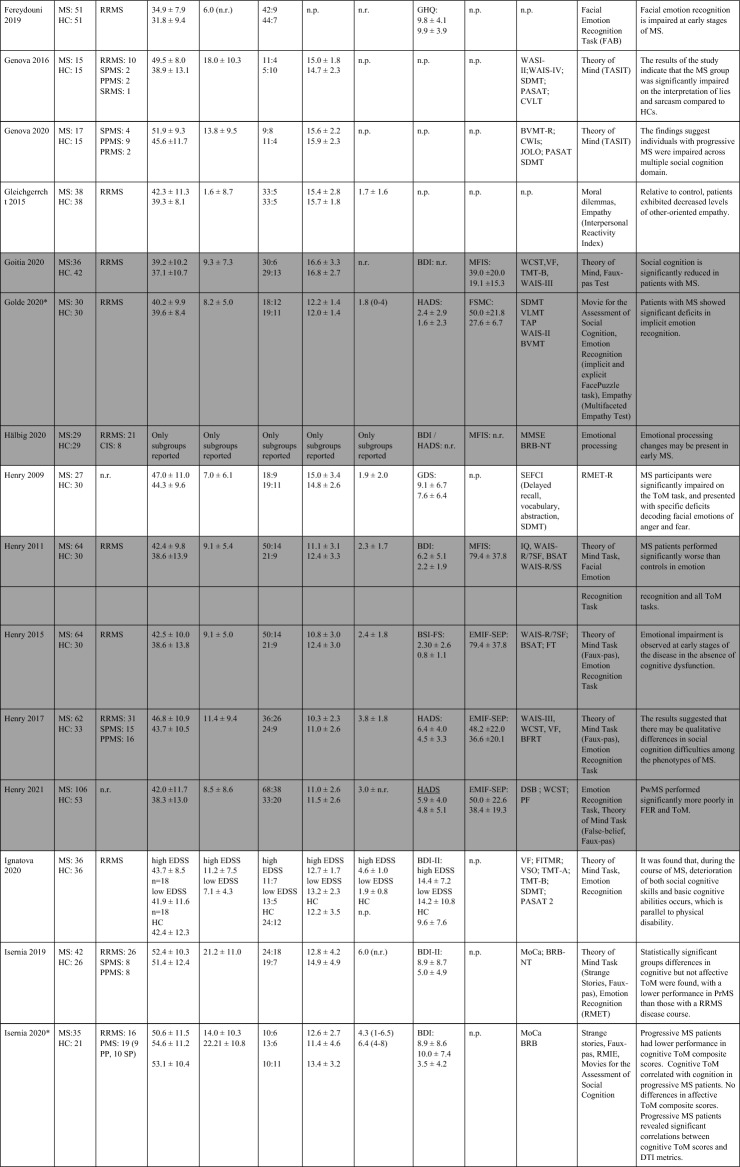

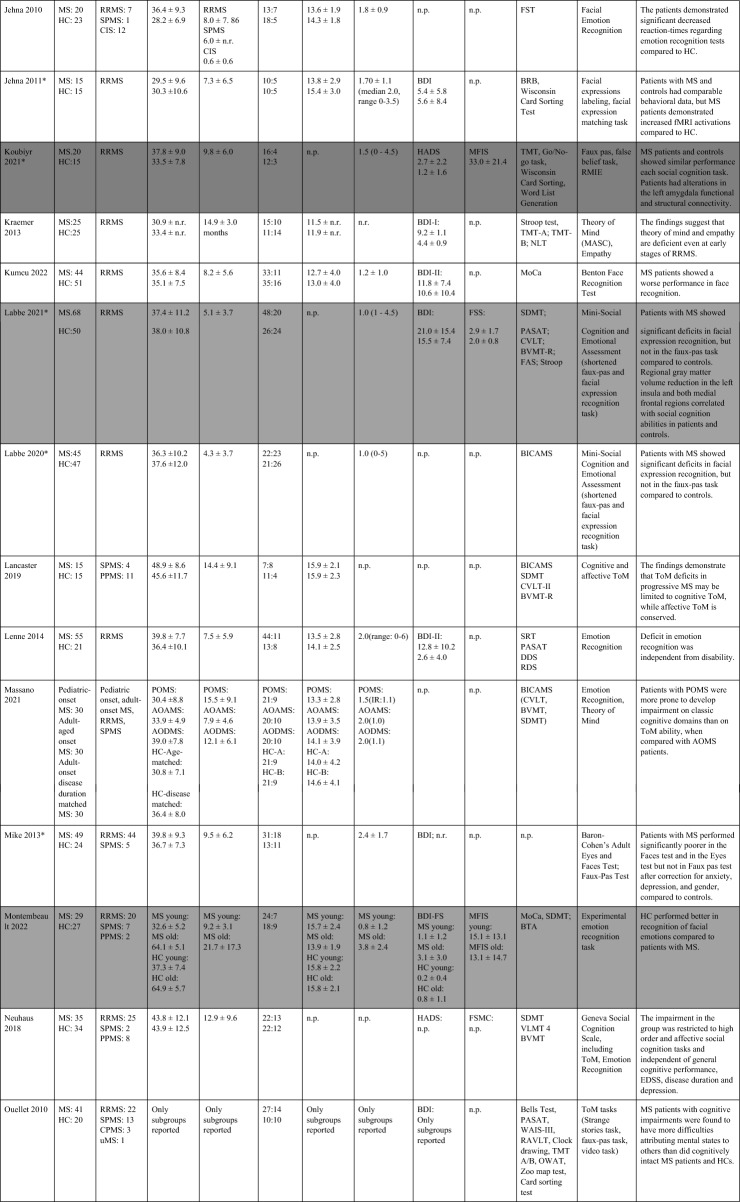

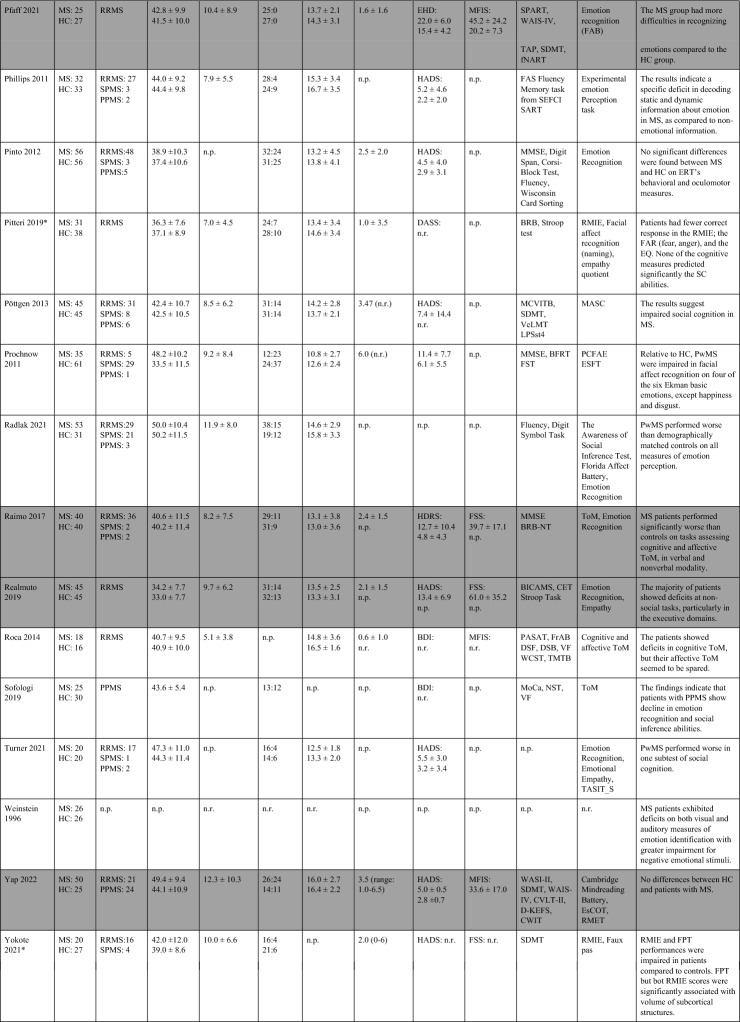
Study overview: describes demographic and clinical characteristics, clinical symptoms, socio-cognitive tasks used and the main results.

Studies shaded in grey provide data on depressive level, fatigue, and cognitive scores of patients with MS.

MS, Multiple sclerosis; HC, Healthy controls; BDI: Beck’s Depression Inventory; RRMS, relapsing–remitting multiple sclerosis; RSPMS, relapsing secondary progressive multiple sclerosis; EDSS, Expanded Disability Status Scale; n.r. , not reported; IQ, intelligence quotient; IQR, Interquartile range; MFIS, Modified Fatigue Impact Scale; ADS-L, Allgemeine Depressionsskala-Langform; AIT, Attribution of intentions test; APACS, Assessment of Pragmatic Abilities and Cognitive Substrates; AToM, Affective Theory of Mind; ATT, Advanced Test of ToM; BDI (-FS) , Beck Depression Inventory (-Fast Screen); BERT, behavioral emotion recognition test; BFRT, Benton Facial Recognition Test; BICAMS, Brief International Cognitive Assessment for MS; BRB-A/NT, Brief Repeatable Battery version A/of Neuropsychological Test;BSAT, Brixton Spacial Anticiation Test; BVMT (-R) , Brief Visuospatial Memory Test (-Revised); C&I, Conversation and Insinuation; CI, cognitive impairment; Cimp, cognitively impaired; Cint, cognitively intact; CIS, clinically isolated syndrome; COWAT, Controlled Oral Word Association Test; CPMS, chronic progressive MS; CrT, crossed taping; CSCT, Computerized Speed Cognitive Test; CToM, Cognitive Theory of Mind; CVLT, California Verbal Learning Test; CWIs, Color-Word Intereference subtest; DAGPVT, Dynamic Age and Gender Perception Video Task; DASS, Depression Anxiety Stress Scale; DDS, direct digit span; DEPVT, Dynamic Emotion Perception Video Task; DMT, decision making test; DSB, Digit span backward; DSCT, Digit Symbol Coding Task; DSF, Digit span forward; EAT, Emotion Attribution Task; EDSS, extended disability status scale; EEI, Expressive emotional intensity (adapted from Edman); EET, Emotion Evaluation Test; EMIF-SEP, Échelle modifiée d’Impact de la fatigue-sclérose en plaques; ESFT, Ekman-60-Faces test; FAB, Florida Affect Battery; FAR, Facial Affect Recognition FaFCSR, French adaptation of the Grober and Buschke Free and Cued Selecive Reminding Test; FED, Facial emotion discrimination; FEEST, Facial Expressions of Emotion: Stimuli and Test; FEM, Facial emotion matching; FEN, Facial emotion naming; FER, Facial emotion recognition; FER-FC, FER-forced choice; FER-FR, FER-free recall; FES, Facial emotion selection; FID, Facial identity discrimination; FITMR, Five items 10-min recall; FOFBT, First-order false belief task; FPi/e, FacePuzzle implicit/explicit; FPT, Faux Pas test; FPT-C/A, Faux Pas Test-cognitive/affective; FPT-I/E, Faux Pas Test-Intention/Emotion; FrAB, Frontal Assessment Battery; FSMC, Fatigue Scale for Motor and Cognitive Functions; FSS, Fatigue Severity Scale; FST, Faces Symbol Test; FT, Fluency test; GDS, Geriatric Depression Scale; GeSoCS, Geneva Social Cognition Scale; GHQ, general health questionnaire; G/NG, Go/No Go; HADS, Hospital Anxiety and Depression Scale; HDRS, Hamilton Depression Rating Scale; HC, healthy control; HCMRI, HCs tested in the MRI; HCPC, HCs tested on the PC; HI, Humor identification; IAPS, International Affective Picture System; IPS, Information processing speed; IQR, interquartile range; IRI (EC/PD/PT/F) , Interpersonal Reactivity Index (empathic concern/personal distress/perspective taking/fantasy); JOLO, Judgment of Line Orientation Test; LAOC, Lexical awareness in OC; LAWC, Lexical awareness in WC; LEAS, Levels of Emotional Awareness Scale; LF, letter fluency; LPSst4, Subtest 4 of the Leistungsprüfsystem; LT, labelling test; MASC, Movie for the Assessment of Social Cognition; MASC-T/I/A/C, MASC-Thoughts/Intention/Affective/Cognitive; MCVIT, Multiple Choice Vocabulary Intelligence Test; MFIS, modified fatigue impact scale; Mini-SEA, Mini-Social cognition & Emotional Assessment; MMSE, Mini Mental Status Examination Test; MoCa, Montreal Cognitive Assessment; MS-FS, MS-specific fatigue scale; MS, multiple sclerosis; n.d. , no data; NLT, number-letter task; n.p. , not performed; NST, Number Series test; OC, oral comprehension; PA, Picture Arrangement (subtest of WAIS-III); PASAT (-RA) , (Rao Adaptation of the) Paced Auditory Serial Addition Test; PatMRI, Patients tested in the MRI; PatPC, Patients tested on a PC; PCFAE, Test of Perceptual Competence of Facial Affect Recognition; PF, phonetic fluency; POFA, Pictures of Facial Affect; PPMS, primary progressive MS; PMS, progressive MS; PRMS, progressive-relapsing MS; PwMS, Patients with MS; Q-IDS, Quick Inventory of Depressive Symptoms; RAPM, The Raven’s Advanced Progressive Matrices; RDS, reverse digit span; RMET(-R) , Reading the Mind in the Eyes Test (revised); RRMS, relapsing–remitting MS; RSPMS, relapsing secondary progressive MS; RTET, reaction times for emotion recognition test; RTGender, times for the gender test; RTPPET, reaction times for the Posner paradigm emotional test; SAOC, Syntaxic awareness in OC; SART, Sustained Attention to Response Task; SAWC, Syntaxic awareness in WC; SC, social cognition; SDMT (-RA) , (Rao Adaptation of the) Symbol Digit Modalities Test; SEFCI, Screening Examination for Cognitive Impairment; SET(-Tot) , Empathy Test (Total score); SF, semantic fluency; SI-E, Social Inference-Enriched; SI-M, Social Inference-Minimal; SIFET, Static Images of Facial Emotion Task; SIPPT, Static Identity Perception Photograph Task; SOFBT, Second-order false belief task; SPART (-D) , (delayed recall of the) Spacial Recall Test; SPMS, secondary progressive MS; SRT (LTS/CLTR/D) , Selective Reminding Test (Long Term Storage/Consistent Long Term Retrieval/Delayed recall); SST (MT/PT) , Strange Stories Task (Mental task/Physical task); SST-D/W/M/E, Strange Stories Task-Doublebluff/Whitelie/Misunderstanding/Emotions; TAP, Test Battery of Attentional Performance; TASIT, The Awareness of Social Interference Test; TC, Text comprehension; TMPS, Theory of Mind Pictures Sequencing Test; TMT(A/B) , The Trail Making Test (A/B); ToM, Theory of Mind; (c/e/min/mis) ToM, correct/excessive/minor/missing ToM; uMS, undetermined MS; VAMA (t/c/a) , Virtual Assessment of Mentalising Ability (total/cognitive/affective); VeLMT, Verbal Learning and Memory Test; VELS, Voice Emotion Labeling Subtest of the FAB; VF, Verbal fluency; VLMT, Visual Learning and Memory Test; VPT, visual perspective taking; VRI, Verbal Reasoning Index; VSO, visual spacial orientation; WAIS-IV, Wechsler Adult Intelligence Scale; WAIS-R/DSCS, Digit-symbol coding subtest of the WAIS-R; WAIS-R/7 SF, The Ward seven-subtest of the revised WAIS; WAIS-R/SS, similarities subtest of the WAIS-R; WASI-II, Wechsler Abbreviated Scale Intelligence; WC, written comprehension; WCST, Wisconsin Card Sorting Test; WLG, Word List Generation, Notes: (1) Batista et al.^28,41,43^ used the same data from patients with MS and healthy controls. Data from Batista et al.^41,43^ are expressed in percent, 2018 in points. (2) Carotenuto (2018): The SET-Test was acquired from 33/42 patients. (3) Cecchetto et al.^32^: The FSS was acquired from 26/30 patients. Studies with asterisk (*) provide imaging information and are therefore also included in Table 2.

**Table 2:**
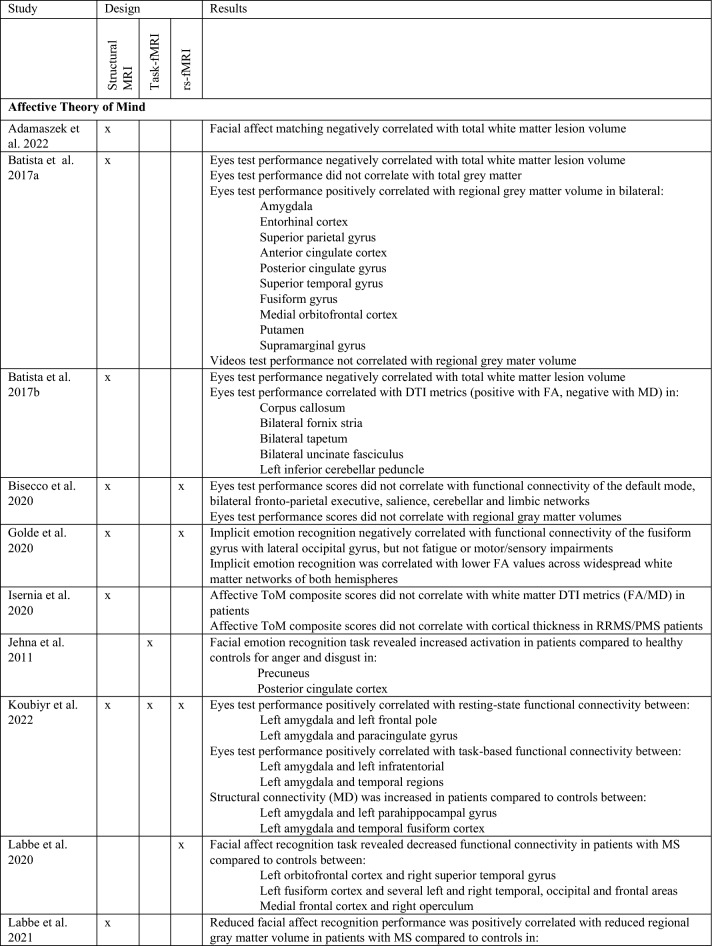

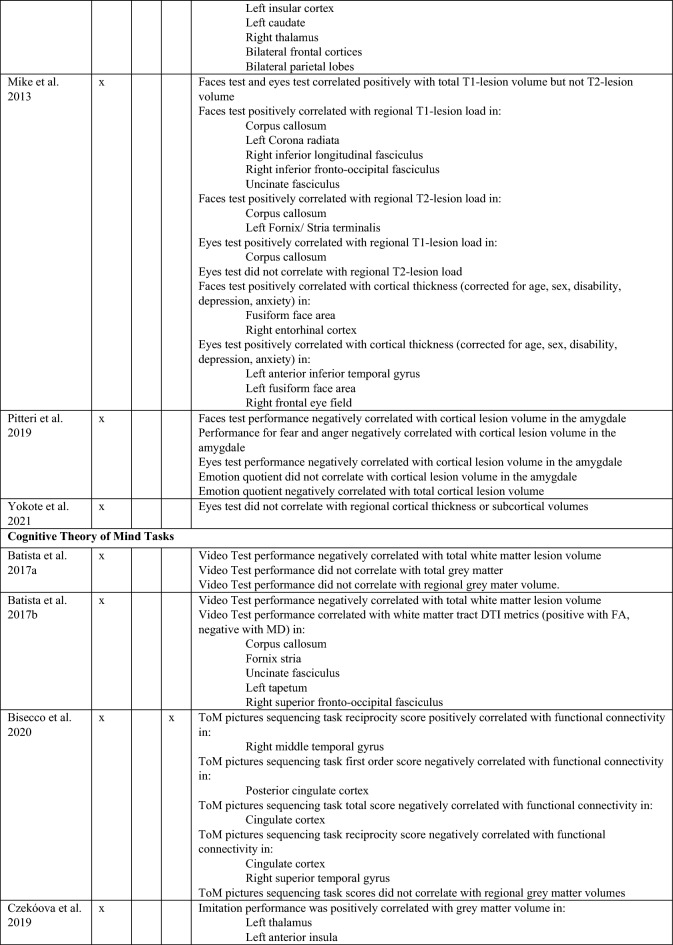

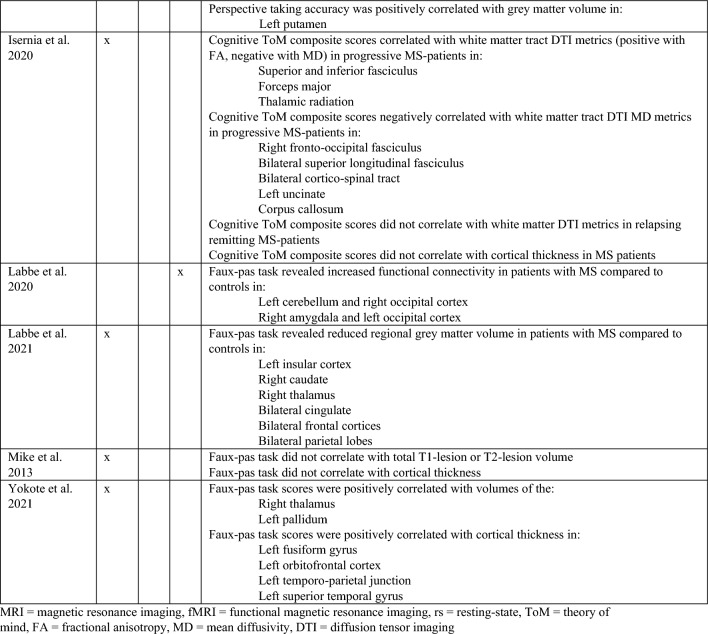
Results of studies that investigated neural correlates of socio-cognitive deficits using structural and functional MRI measures.

MRI, Magnetic resonance imaging, fMRI, Functional magnetic resonance imaging, rs, Resting-state, ToM, Theory of mind, FA, Fractional anisotropy, MD, Mean diffusivity, DTI, Diffusion tensor imaging.

### Included studies

In the following paragraphs, the results of the systematic review will be presented (1.1) as well as the systematic review results of studies including data on cognition, depression, and fatigue (1.2). In the following, the risk of bias analysis will be presented (1.3) and the results of the meta-analyses (1.4), which are divided in results for the analysis of cToM (1.4.1) and aToM (1.4.2). These results are followed by on overall sensitivity analysis (1.5). In a second step, we investigated the impact of depression, fatigue and cognitive status on socio-cognitive impairment in a meta-regression (2) and present the results on the neural correlates of socio-cognitive decline (3), again separately for aToM (3.1) and cToM (3.2).

#### Systematic review

Table 1 provides an overview of all included studies detailing demographic and clinical sample characteristics, socio-cognitive tasks used and the main results. Overall, 58 studies that investigated SC in patients with MS were included. Of those, all except for two studies^17,18^ provided information on the type of MS. The majority of patients were diagnosed with relapsing–remitting MS (RR-MS). MS is generally more prevalent in women^19^, which is also reflected in skewed sex distributions of the included study samples (with one exception^20^).

All except one study^21^ used ToM as one of their outcomes. Cognitive ToM was assessed in 31 studies and assessed using either variations of the faux-pas task (a task comprising a situation/context where one character (the speaker) makes a statement that is unintentionally offensive to the listener because the speaker has a false belief), a false-belief task (two different types: first-order false-belief tasks involve attribution about other’s false belief with regard to real events; second-order false-belief tasks assess what people think about other people’s thoughts), a strange stories task (a selection of stories that test pretense, jokes, lies, white lies, misunderstandings, persuasion, appearance/reality, figures of speech, irony, double bluffs, contrary emotions, and forgetting) or a video test (a series of videotaped vignettes of social scenarios followed by questions about thoughts, feelings, and/or intentions of the characters). Cognitive TOM was found to be impaired in patients with MS in 26 of 31 studies. Affective ToM was measured in 52 studies using different types of emotion recognition or processing tasks, in which either the eyes or the whole face of a person were presented. Participants were then asked to label the emotion represented by the eyes/face. 42 out of 52 studies showed an impairment in emotion recognition and/or emotion processing in MS patients compared to healthy controls. Two studies investigated VPT^22,23^, both showing significantly reduced imitation and poorer accuracy in perspective taking than healthy controls. SDM was investigated in two studies^21,24^ using either a moral/conventional distinction task (in which social situations and moral and conventional transgressions were presented, as well as an authority jurisdiction)^24^, or vignettes consisting of moral dilemmas (e.g. choice of whether or not to harm a person to save five other people)^21^. Results showed a lack of distinction between conventional and moral judgement in patients with MS^24^, as well as a reduced moral permissibility in patients with MS^21^.

#### Systematic review of studies including data on cognition, depression, and fatigue

Notably, even though information on the level of depressive symptoms, fatigue, and degree of cognitive impairment are essential for treatment decisions, only 18 out of 58 studies reported data on all three domains. All of them showed higher fatigue and depressive scores in patients with MS compared to healthy controls. In all studies, depression was described as mild because patients with severe symptoms were generally excluded. Several cognitive domains were reported as impaired in patients in MS compared to healthy controls, including executive functions^25,26^, semantic fluency^27^, and IQ scores^27^. Three studies^17,28,29^ included depressive and fatigue scores as control variables in their analyses; two of them found that even after controlling for these variables, patients with MS performed significantly worse on aToM^28^ and cToM tasks^28,29^, one study found no significant influence of these variables on impaired ToM performance^17^. Two studies showed a significant negative correlation between aToM performance and depressive symptoms (higher depressive scores were associated with lower aToM scores)^1,30^. Only one study did not demonstrate correlations between socio-cognitive measures and depression^31^. Three studies showed a significant positive correlation between aToM and cognition (higher aToM scores were associated with better performance on long-term storage and retrieval tasks, the Symbol-digit-modalities test^32^; and semantic fluency and IQ^27^). Reduced performance in ToM correlated with poorer executive function, intellectual ability and episodic memory^31^, and cToM and IQ and semantic fluency^27^. Pfaff et al. showed that inhibition and divided attention measures were predictive of difficulties in identifying facial emotions (aToM) in MS patients^33^, Montembeault et al. reported a positive correlation between general cognition (Montreal Cognitive Assessment, MoCA), but not with attention, measured with the Brief Test of Attention, the Symbol-Digit-Modalities Test, or the Stroop Inhibition task^34^.

#### Risk of bias assessment

Results of the RoB Assessment are shown in Table 3. Overall, only one domain was rated with a low RoB in all studies concerning the assessment of the study outcome, namely “Can we be confident in the assessment of the outcome?”. Most studies did not match their groups for possible confounders (e.g., comorbid depression) or controlled for possible confounding variables, leading to either an unsure RoB when no data was provided or to high RoB in cases were confounders were not assessed.

**Table 3:**
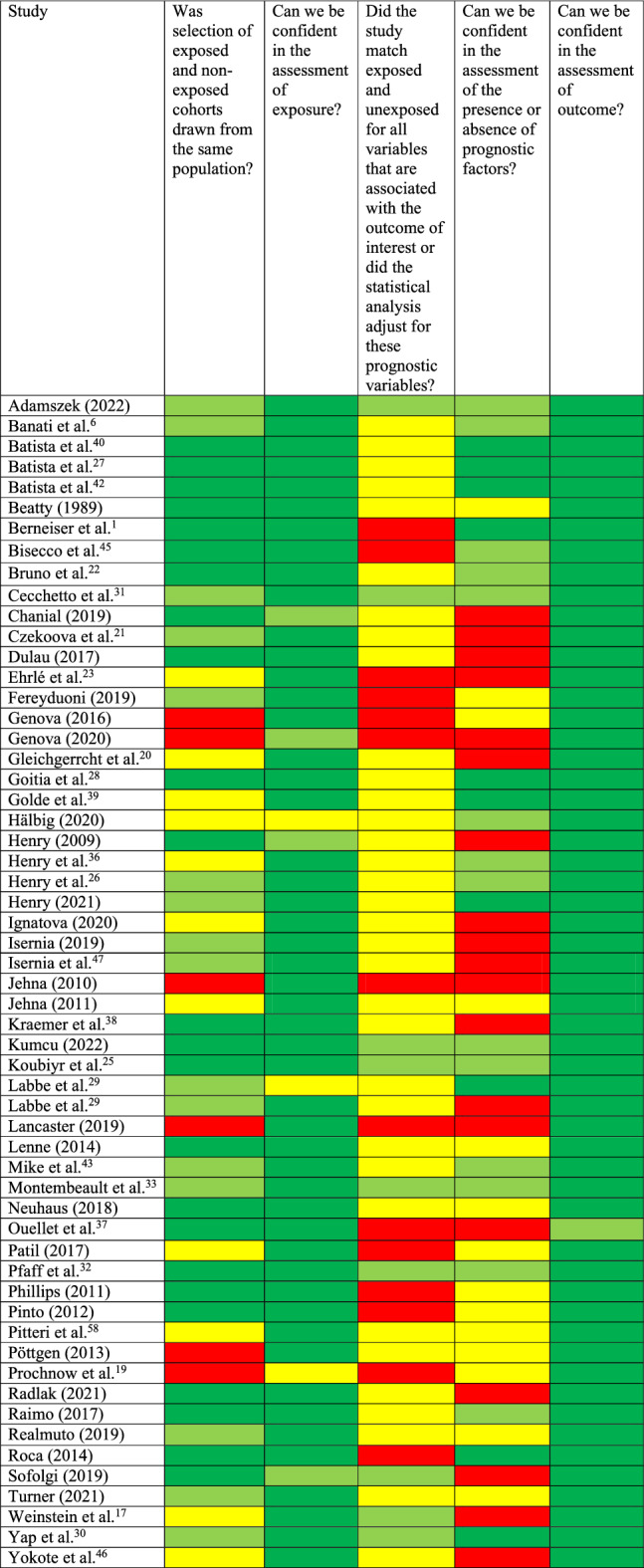
Risk of bias assessment.

Red color indicates a high risk of bias, yellow color indicates a medium risk of bias, green color indicates a low risk of bias (dark green: no concerns, light green: small concerns, but still a low risk of bias), assessed with the “Tool for Assessing Risk of Bias in Cohort Studies” by the CLARITY Group^60^.

#### Meta-analyses

Meta-analyses were only possible for the primary outcome (ToM), because there was not enough data available for the remaining socio-cognitive domains (SDM, VPT). Due to the high heterogeneity of outcomes and ToM constructs, we decided to calculate separate analyses for cToM (Results of Faux-Pas Tests and results of Video tests) and aToM (often labelled as emotion recognition). Furthermore, if applicable, we subdivided the meta-analysis in the respective tests used for assessment to ensure better comparability.

### Cognitive theory of mind

#### Faux-Pas test

**Figure 2:**
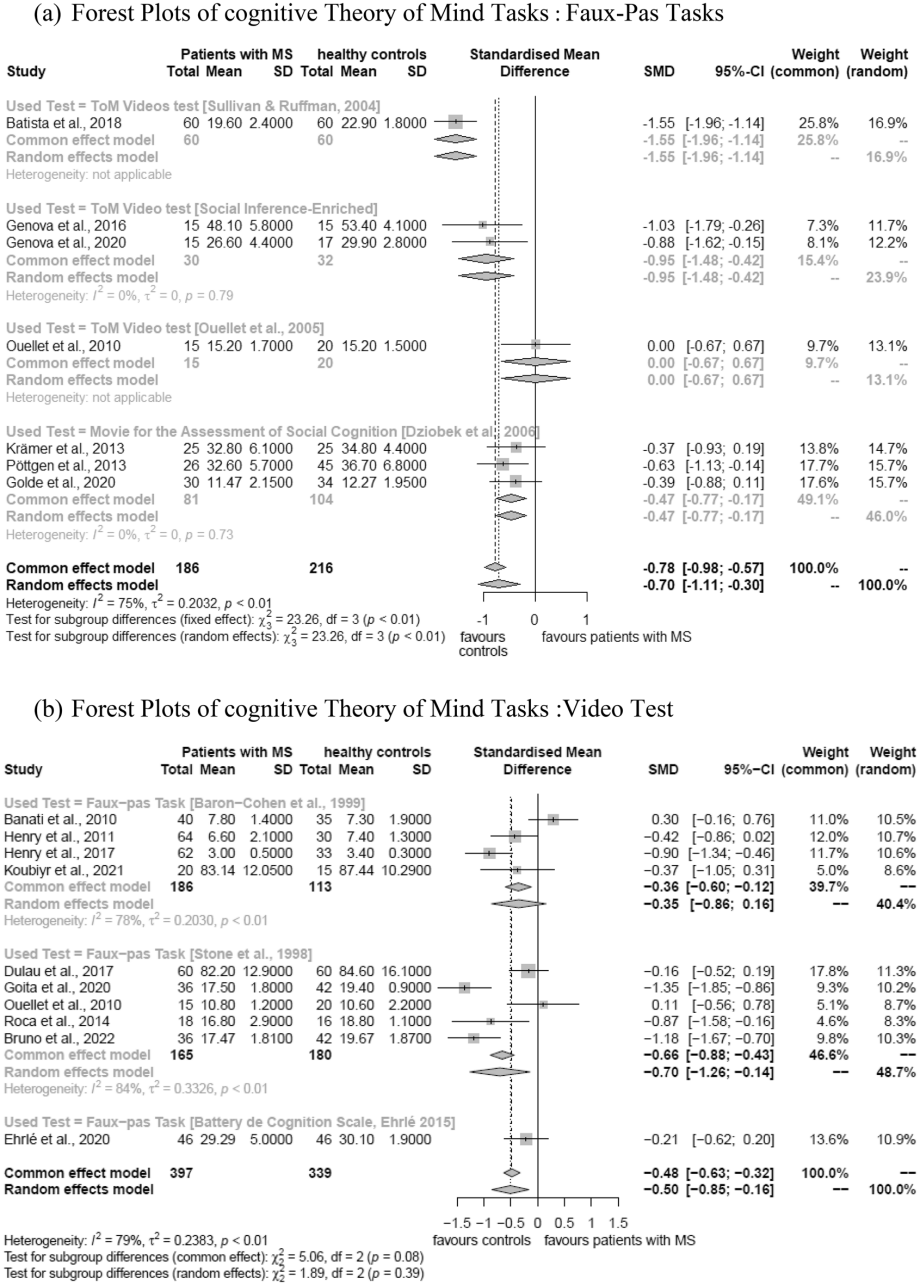
Forest plots of cognitive theory of mind tasks.

Overall, ten studies were included in the meta-analysis that included variations of the faux-pas test and compared patients with MS and healthy controls. Four used the faux-pas task by Baron-Cohen et al.^35^, five the faux-pas task by Stone et al.^36^, and one study using a faux-pas task included in a Social Cognition battery. Results showed that healthy controls performed significantly better than MS patients across the different tasks: SMD = (− 0.50), 95% CI (− 0.85) to (− 0.16), I^2^ = 79%) and also in each of the two task versions where sufficient data was available for separate analyses (Baron-Cohen task: SMD = (− 0.35), 95% CI − 0.86 to 0.16, I^2^ = 78%; Stone Task: SMD = (− 0.70), 95% CI − 1.26 to 0.14, I^2^ = 84%). A forest plot for the outcome is displayed in Fig. 2a.

#### Video test

Seven studies were included that investigated differences in performance using four different types of ToM Video Tests between healthy controls and patients with MS. Overall results are reported, because there was not enough data available to consider individual outcomes. The overall result indicates that healthy controls performed significantly better on Video Tests than patients with MS (SMD = (− 0.70), 95% CI (− 1.21) to (− 0.30), I^2^ = 75%). The forest plot for the outcome is displayed in Fig. 2b.

### Affective theory of mind/emotion recognition

**Figure 3:**
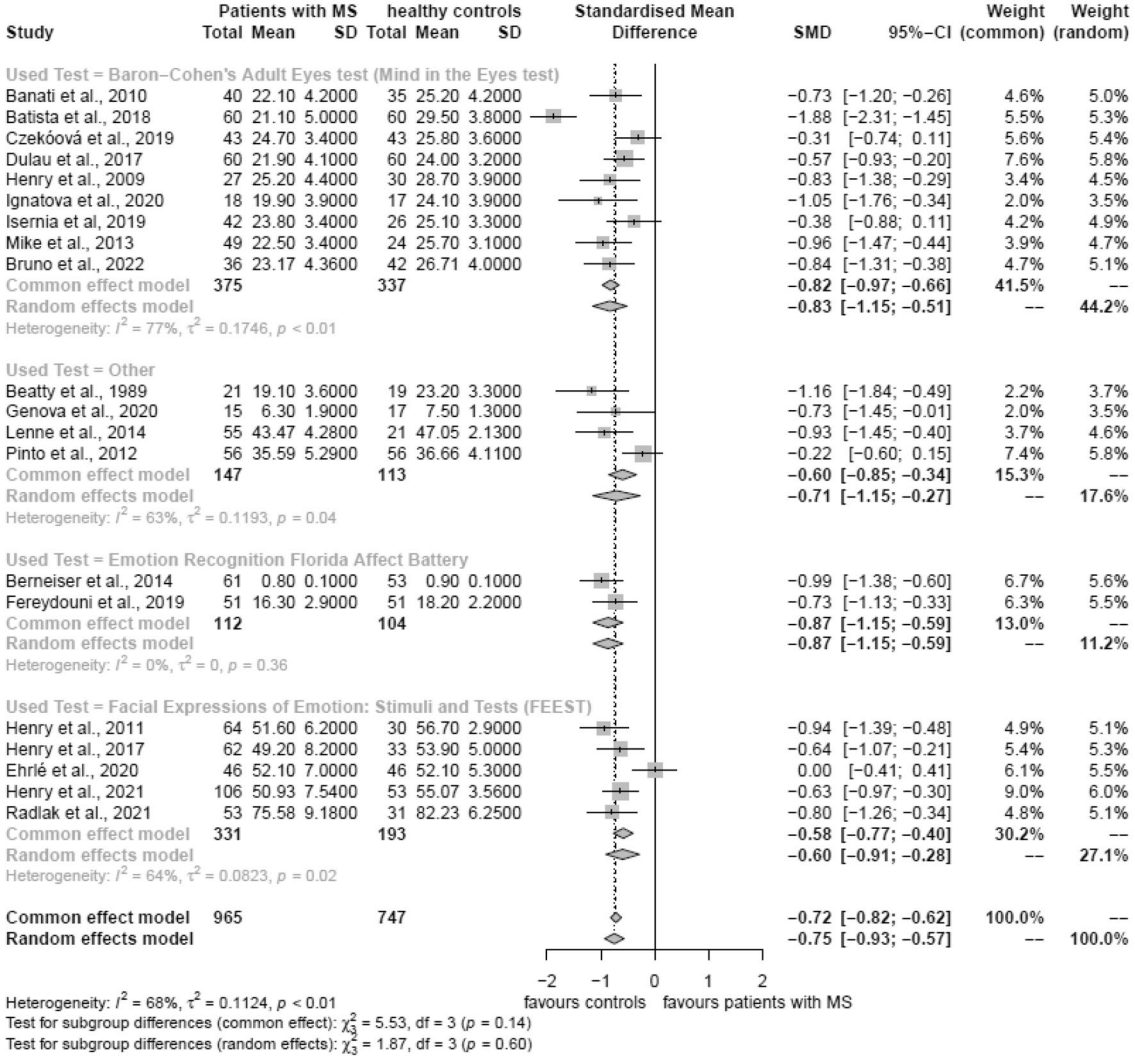
Forest plots of affective theory of mind tasks.

Twenty studies were included in the meta-analyses that investigated aToM tasks. The overall effect size of the random effects analysis was − 0.75(CI:(− 0.93) to (− 0.57), favoring healthy controls. Nine studies used the Baron-Cohen Adult Eyes Test, showing better performance in healthy controls compared to MS patients (SMD =  − 0.83, 95% CI (− 1.15) to (− 0.51), I^2^ = 77%). Two studies used the Emotion Recognition Florida Affective Battery (SMD =  − 0.87, 95% CI (− 1.15) to (− 0.59), I^2^ = 0%); four studies used the Facial Expression of Emotion (FEEST) Tests (SMD =  − 0.55, 95% CI (− 0.93) to (− 0.17), I^2^ = 71%) and four studies used similar experimental emotional recognition tasks that were grouped together as they all used different tests (SMD =  − 0.71, 95% CI (− 1.15) to (− 0.59), I^2^ = 0%), all of them showing a better performance in healthy controls than patients with MS. The forest plot is displayed in Fig. 3.

### Sensitivity analyses

The results of the sensitivity analyses using fixed effects models are displayed in Figs. 2a,b and 3 and did not show any significant differences compared to the random effects models.

### Meta-regression analyses: Impact of depression, fatigue and cognitive status on socio-cognitive impairment

Meta-regressions analyses could be conducted for cToM(faux-pas), cTom(videos), and aToM, see Table 4. Due to the substantial heterogeneity of assessments that were used to quantify cognitive impairment, fatigue and depression, as well as insufficient data, we were not able to integrate the variable cognition, as well as depression for cToM(faux-pas) and fatigue for cTom(videos). In the analysis on cToM(faux-pas), that included three studies^27,29,37^, R^2^ was 100% (meaning 100% of the difference in true effect sizes can be explained by the predictor fatigue), and the intercept as well as the predictor fatigue were significant (intercept: − 1.73 (*p* < 0.001), fatigue: 0.02 (*p* < 0.05)). This means that performance on cTom (faux-pas) decreased with higher fatigue levels. In the meta-regression on cToM(video), four studies were included^28,38–40^. Yet, the overall model did not reach significance (R^2^ = 0%), so the predictor depression did not explain any additional variance in the results. Five studies that investigated aToM were included^1,17,22,27,37^. The intercept (the difference in performance between healthy controls and patients with MS when all predictors have a value of 0) was significant (− 1.82, *p* < 0.05) and favored healthy controls, similar to the other results. Both predictors, depression (0.13), and fatigue (0.01) did not reach significance. The overall R^2^ of the model was 28.73% and the I^2^ was at 32.76%, indicating only low heterogeneity.

**Table 4:** Results of meta-regressions.

Models	cToM	cToM	aToM
	Faux-Pas test *k* = 3	Video test *k* = 4	Emotion recognition *k* = 5
	Estimate (SE)	Estimate (SE)	Estimate (SE)
Intercept	− **1.73 (*p* < .001)**	− 1.03 (*p* = .188)	− **1.82 (*p* < .05)**
Depression	–	0.05 (*p* = .527)	0.13 (*p* = .111)
Fatigue	**0.02 (*p* < .05)**	–	0.01 (*p* = .256)
Cognitive status	–	–	–
I^2^/R^2^	0%/100%	88.62%/0.00%	32.76%/28.73%

For the analysis of cToM: Faux-Pas test, only three studies could be included providing data on fatigue due to missing data in the other eligible studies and possible moderators. Goita et al. (2020) used the MFIS to assess fatigue, Henry et al.^27,37^ used the EMIF-Sep to assess fatigue. For the analysis cToM: Video Test, we could include four studies providing data on depression. Batista et al.^28^, Ouellet et al.^38^ and Krämer et al. (2013) used Beck’s Depressive Inventory (BDI) to assess depression, Golde et al.^40^ used the Hospital Anxiety and Depression Scale: Subscale Depression (HADS-D). In the analysis aToM: Emotion Recognition, we could include five studies providing data on depression and fatigue. Three studies used the BDI and the MFIS^1,22, 37^, and two studies used the HADS-D and the EMIF-Sep^27^, Henry et al. (2021) to assess depressive symptoms and fatigue. Interpretation: I^2^ = after inclusion of the predictor, XX% of the variability in our data can be attributed to the remaining between-study heterogeneity. R^2^ = XX% of the difference in true effect sizes can be explained by the predictor. Intercept = The difference in the outcome variable between our two groups when all integrated predictors have a value of 0.

Significant values are in [bold].

### Neural Correlates of socio-cognitive decline

Fourteen studies provided information on the neural correlates of socio-cognitive impairment in MS. Among those, thirteen studies investigated aToM, nine studies investigated cToM (for a detailed overview please see Table 2).

#### aToM

Eleven studies used structural MRI to investigate neural correlates of aToM using a number of different outcomes (e.g., eyes or faces tests, aToM composite scores). Of those, four studies demonstrated positive correlations between total white matter lesion volume and aToM performance^41–44^, one with total grey matter volume^43^. Using more regional approaches, two studies revealed association between aToM and white matter lesions^44^ or other DTI derived metrics (e.g., fractional anisotropy, mean diffusivity^41^), highlighting the potential role of disconnection of regions associated with socio-cognitive processing. Six studies that used voxel-based morphometry or assessed regional cortical thickness demonstrated positive correlations between aToM and integrity of the amygdala, fronto-temporal and other regions^26,28,44,45^, whereas two studies could not confirm these findings^46,47^.

Four studies used task-free resting-state functional imaging^26,30,40,46^ and three of these studies highlighted the contribution of frontal and temporal networks to aToM impairment in MS. Specifically, they demonstrated a negative correlation between implicit emotion recognition performance and functional connectivity of the fusiform gyrus with lateral occipital gyrus^40^, a positive association between RMET performance and functional connectivity between the left amygdala and frontal pole/paracingulate cortex^26^, or decreased functional connectivity between fronto-temporal regions in patients compared to controls during a facial affect recognition task^30^. On the other hand, Bisecco et al.^46^ did not find any correlations between the RMET performance and functional connectivity of the default mode, bilateral fronto-parietal executive, salience, cerebellar and limbic networks.

#### cToM

Eight out of nine studies investigated neural correlates of cToM using structural MRI. Three studies investigated the association to the total white matter lesion volume, with two studies demonstrating a negative association between the lesion volume and the video test performance^28,43^, and one study without an association between the lesion volume and the faux-pas task ability^44^. One study failed to show a significant correlation between cToM performance and total grey matter volume^28^.

Regional grey matter volume reductions were investigated in seven studies. Of those, three studies demonstrated a significant correlation between reduced volume of the thalamus and reduced cToM performance^22,45,47^ and additional positive correlations between grey matter volumes in different cortical regions (e.g., insula, frontal cortex, temporal and parietal cortex) and cToM. Four studies did not find any association between performance and regional grey matter integrity^43,44,46,48^. Two studies demonstrated an association between altered white matter DTI metrices (i.e., reduced FA and higher MD), especially in the corpus callosum and the superior fasciculus^41,48^. Two studies used resting-state fMRI and found that increased functional connectivity between the occipital cortex and the cerebellum/amygdala in MS-patients compared to controls was correlated with better cToM performance^30^, as well as positive and negative correlations between different subscales of a cToM picture sequencing task and functional connectivity of the right middle temporal and (posterior) cingulate cortex^46^.

Four imaging studies provided information on depression, fatigue and cognition, with three studies investigating aToM^26,40,45^, and two cToM^22,45^, but used different measures to quantify depression, fatigue and cognition, that complicate meaningful comparison of the results.

## Discussion

The present systematic review and meta-analysis confirms previous reports demonstrating socio-cognitive impairment in patients with MS^9–11^ and suggests that comorbid cognitive and affective symptoms or fatigue can further exacerbate these impairments. The vast majority of eligible studies investigated different aspects of ToM and approximately 80% of the included studies reported impairment of either affective or cognitive ToM in MS patients relative to healthy control groups. Our meta-analyses demonstrated more pronounced impairment for aToM (ES = 0.8) compared to cToM (ES = 0.05-0.07). This pattern is in line with results of previous meta-analyses, that also demonstrated more pronounced impairment of aToM, especially for the RMET and facial emotion recognition tasks, compared to cToM (i.e., faux-pas tasks)^9,11^. While only 4/58 eligible studies investigated different aspects of social cognition in MS (VPT/SDM), all of them reported significant impairment compared to healthy control groups. This highlights the need to further investigate other socio-cognitive processes than ToM in MS and to determine the potential interplay with other clinical symptoms (i.e., depression, fatigue or cognitive status). This was not possible in the present study, due to the small number of available studies.

Eighteen of the included studies provided additional information on clinical symptoms that may impact on socio-cognitive impairment, but only three studies controlled for these variables in their analyses^17,27,28^. Nonetheless, the overall pattern of results from individual studies suggests that depression, fatigue and cognitive impairment can contribute to socio-cognitive impairment in MS. This was further supported by the results of our meta-regression analyses that demonstrated a specific contribution of fatigue to the degree of impairment in cToM, but not aToM. However, future research is needed, to systematically investigate whether specific clinical symptoms exacerbate the degree of impairment in different aspects of socio-cognitive functioning and to determine causal relationships between them.

It needs to be acknowledged that the results of this study are based on a relatively small number of studies and are therefore to be interpreted with caution. Nonetheless, our study included about 30% more studies compared to the most recent previous meta-analysis by Lin et al.^11^. Two earlier meta-analyses published in 2016 included only about half the number of studies^9,10^, which highlights an emerging interest in this topic. This not unsurprising, because intact social functioning has been linked to relationship and vocational success, and better life satisfaction in healthy individuals^49^. Moreover, socio-cognitive impairment can have a profound negative impact on social participation, resulting in loneliness and poor mental health^50^, which may be particularly detrimental in individuals attempting to cope with progressive conditions like MS. Nonetheless, the direct contribution of socio-cognitive impairment to reduced quality-of-life (QoL) in MS is currently unclear. For example, while Philips et al.^51^ demonstrated that emotion regulation capacity was positively correlated with higher QoL in MS patients, others failed to demonstrate independent contributions of socio-cognitive impairment to QoL (e.g.^52^). Such discrepancies are likely explained by mutual interdependencies of both social cognition and QoL with clinical symptoms that are frequent in MS^53^. However, only about ~ 30% of our included studies reported information on specific socio-cognitive outcomes and (substantially varying degrees of) cognition, fatigue and depression. Only three studies controlled for these symptoms in their analyses, none reported associations with QoL. Thus, future systematic research is needed to disentangle the complex interactions between socio-cognitive impairment and cognition, fatigue and depression, and how they affect real-life outcomes, including QoL or the ability to cope with disease progression.

Finally, the systematic review of the anatomical and functional brain correlates underlying socio-cognitive impairment in MS revealed substantial heterogeneity between studies with regard to characteristics of the included patients, imaging methods, and outcome measures. As for the behavioral studies described above, the majority of imaging studies focused on different aspects of ToM (cTOM: 9 studies, aTOM: 13 studies). With regard to imaging methods, twelve studies used structural imaging and investigated global or regional grey and white matter changes. Only five studies employed functional MRI. Despite partially conflicting findings, these studies demonstrated that lesions affecting major cortical or subcortical hubs (e.g., orbito-frontal or insular cortex, the amgydala) within task-relevant regions of the “social brain”^54^ or domain-general networks (e.g., ventral/dorsal attention, salience or default networks) can be related to the degree of specific socio-cognitive impairment. Similarly, several studies demonstrated the contribution of white matter pathways (e.g., corpus callosum, uncinate fasciculus, superior longitudinal fasciculus) or functional connectivity changes in specific networks to socio-cognitive impairment. However, aside from the general heterogeneity (including paradigms, methods for data acquisition and analyses, patient characteristics, etc.), the interpretation of neural findings in the included studies is often further complicated because the observed local or network level findings partially overlap with those reported in the much more extensive literature on neural underpinnings of cognition, depression and fatigue in MS^3,55^. Moreover, only four imaging studies provided additional information about these potentially conflicting variables, which were also not directly related to the imaging results. Therefore, results of individual studies need to be interpreted with caution.

In sum, the present study demonstrates substantial impairment of socio-cognitive processes in MS and highlights the potential mediating role of comorbid clinical symptoms. We identify several current evidence gaps and larger scale studies using comprehensive and coordinated assessments of socio-cognitive parameters (e.g., similar to current efforts for establishing core outcome parameters for clinical trials, https://www.comet-initiative.org/), potential mediators and neural correlates are urgently needed.

## Methods

The present systematic review and meta-analysis followed the Preferred Reporting Items for Systematic Reviews and Meta-Analyses (PRISMA) guideline^16^. The pre-registered review protocol can be assessed at www.crd.york.ac.uk/PROSPERO/ (ID: CRD42020206225).

### Systematic search, study selection and eligibility criteria

A systematic electronic search was conducted in MEDLINE Ovid, Web of Science Core Collection, CENTRAL, and PsycInfo up to 31st August 2020 with the following keywords: multiple sclerosis, theory of mind, mind reading, social cognition, social cognitive deficits, emotional expression, facial emotion, empathy, social decision making. An update search was conducted on the 15th December 2022. Our search string for MEDLINE Ovid is provided as an example in Supplementary Table 1.

Three review authors (MR, LG, LH) screened all obtained titles and abstracts according to pre-defined criteria using the Covidence Software (https://www.covidence.org/). Full-texts were again screened for studies meeting the inclusion criteria. Disagreements between the reviewers were solved by discussion.

We included studies that investigated social cognition in male and female patients ≥ 18 years old with multiple sclerosis diagnosis (all diagnostic types) compared to a healthy control group. We defined ToM as our primary outcome, because ToM is a key aspect of social cognition, adequate ToM performance is critical for establishing proper social interaction and also relevant for coping with chronic conditions such as MS^56^. ToM is defined as the ability to attribute mental states to others or the ability to understand and predict others’ behaviour based on their mental states and is the most frequently studied socio-cognitive process across development and in healthy and pathological aging. Please note, separate meta-analyses were calculated for cToM and aToM to reduce heterogeneity and because both are supported by partially different neural networks^57^. Secondary outcomes were chosen to represent two additional major socio-cognitive domains: social perception (recognizing others as “living persons” via the analysis of perceptual information including e.g. visual perspective taking), and social decision-making (using the obtained social information for social decision making)^15^. All socio-cognitive outcomes needed to be tested with standardized tests to be included in our review. If more than one assessment was conducted, only the first timepoint was considered. Studies specifically assessing empathy were not considered because of the highly heterogeneous nature of this concept (e.g., different aspects of empathy are associated with different neural networks) and overlap with emotion processing and ToM^58^.

### Data extraction

Three review authors [MR, LG, LH] extracted the data using a study specific, standardized data extraction sheet. Disagreements were discussed with all authors until consensus was reached. We contacted *n* = 13 authors for missing data. Only four replies were received, two authors provided data^1,59^.

### Quality assessment

We assessed risk of bias (RoB) for each included study using the first six signaling questions of the “Tool for Assessing Risk of Bias in Cohort Studies” by the CLARITY Group^60^. Signaling questions can either be answered with “definitely yes” (low RoB), “probably yes”, “probably no”, “definitely no” (high RoB). Note, that three signaling questions were not applicable to our research question and studies. Two review authors (LG, LH) individually assessed RoB for each study. If no consensus could be reached, a third author (MR) was involved.

### Meta-analyses

We conducted random-effects pairwise meta-analyses to investigate the degree of SC impairment in patients with MS relative to healthy controls. Data was clustered according to our four outcomes aToM, cToM, SCD and VPT. For each outcome, we also clustered studies according to the tests that were used for assessments (e.g., aToM: Baron-Cohen’s Adult Eyes Test, Emotion Recognition Florida Affective Battery, and the Facial Expression of Emotion Test, FEEST). Meta-analyses were only calculated if *n* ≥ 3 studies were available.

Data analysis was conducted using R. For all analyses, the alpha level was set at 0.05. Standardized mean differences (SMD) were used as effect sizes, because constructs (e.g., ToM) were assessed with different tests. The mean score of the dependent variable, the mean standard deviation, and the number of included participants in each group were used to calculate SMD.

To address heterogeneity, we used the I^2^ statistic. As recommended in the Cochrane Handbook for systematic reviews of interventions^61^, heterogeneity was interpreted as: 0–40%: not important/low heterogeneity; 30–60%: moderate heterogeneity; 50–90%: substantial heterogeneity; 75–100%: considerable heterogeneity. A funnel plot for identifying possible publication bias was calculated in analyses including ≥ 10 studies. Sensitivity analysis were calculated using fixed effect models to control for small-study effects. If the effect estimates of both, the fixed and random effects model are similar, then any small-study effects have little effect on the effect estimate.

To further assess the impact of cognitive status, depressive symptoms, and fatigue on socio-cognitive abilities in patients with MS, meta-regression analyses were conducted using aToM and cToM as outcome variables and cognitive scores (measured via the neuropsychological test that was most frequently reported in the included studies) and depressive symptoms and fatigue (both measured with standardized questionnaires) as possible predictors. Meta-regressions on SDM and VPT could not be conducted as there was not enough data reported in the studies (Note: this analysis requires correlations between the investigated outcome variable and all possible predictors that are included in the model).

### Supplementary Information

Supplementary Information 1.

Supplementary Information 2.

## Data Availability

All data generated or analyzed in this study are included in the published article [and its supplementary information files]. Aggregated data can be shared by the corresponding author on reasonable request.

## Code availability

Code can be shared by the corresponding author on reasonable request.
