# Supplementary material for: A systematic review and meta-analysis of socio-cognitive impairments in multiple sclerosis
Source: Sci Rep. 2024 Mar 26;14:7096. doi: 10.1038/s41598-024-53750-5 (PMC10963773; doi:10.1038/s41598-024-53750-5)
Supplement: Supplementary file 1 — Supplementary Information 1. [file 41598_2024_53750_MOESM1_ESM.docx]

Supplementary Material:

**Social cognition in multiple sclerosis: Systematic review and meta-analysis of impairment, relation to clinical symptoms and MRI findings**

Mandy Roheger^a,b^, Lydia Grothe^a^, Laura Hasselberg^a^, Matthias Grothe^a1^, &

Marcus Meinzer^a1^

*^a^ Department of Neurology, University Medicine Greifswald, Greifswald, Germany*

*^b^ Ambulatory Assessment in Psychology, Department of Psychology, Carl von Ossietzky University Oldenburg, Oldenburg, Germany*

*^1^ contributed equally*

**Supplementary Table 1:** Search string for MEDLINE Ovid

| (multiple sclerosis OR MS OR Encephalomyelitis disseminata NOT |
| --- |
| (dementia OR autism OR psychosis OR epilepsy OR ADHD OR schizophrenia OR systematic review OR metaanalysis)) |
| AND (Theory of mind OR mind reading OR social cognition OR social cognitive deficits OR social perception OR emotion recognition OR visual perspective taking OR mental state reasoning OR social functioning OR social cognitive impairment OR emotional expression OR facial expression OR facial emotion OR social perception of emotions OR empathy OR affective prosody OR social decision making OR social interaction) |
