# Supplementary material for: A systematic review and meta-analysis of socio-cognitive impairments in multiple sclerosis
Source: Sci Rep. 2024 Mar 26;14:7096. doi: 10.1038/s41598-024-53750-5 (PMC10963773; doi:10.1038/s41598-024-53750-5)
Supplement: Supplementary file 2 — Supplementary Information 2. [file 41598_2024_53750_MOESM2_ESM.docx]

Adamaszek, M., Krüger, S., Kessler, C., Hosten, N., & Hamm, A. (2022). Clinical and neurophysiological patterns of impairments to emotion attention and empathy in multiple sclerosis. *Journal of Integrative Neuroscience*, *21*(1), 7.

Banati, M., Sandor, J., Mike, A., Illes, E., Bors, L., Feldmann, A., ... & Illes, Z. (2010). Social cognition and theory of mind in patients with relapsing‐remitting multiple sclerosis. *European Journal of Neurology*, *17*(3), 426-433.

Batista, S., d’Almeida, O. C., Afonso, A., Freitas, S., Macário, C., Sousa, L., ... & Cunha, L. (2017). Impairment of social cognition in multiple sclerosis: Amygdala atrophy is the main predictor. *Multiple Sclerosis Journal*, *23*(10), 1358-1366.

Batista, S., Alves, C., d’Almeida, O. C., Afonso, A., Félix-Morais, R., Pereira, J., ... & Cunha, L. (2017). Disconnection as a mechanism for social cognition impairment in multiple sclerosis. *Neurology*, *89*(1), 38-45.

Batista, S., Freitas, S., Afonso, A., Macário, C., Sousa, L., Cunha, L., & Santana, I. (2018). Theory of mind and executive functions are dissociated in multiple sclerosis. *Archives of Clinical Neuropsychology*, *33*(5), 541-551.

Beatty, W. W., Goodkin, D. E., Weir, W. S., Staton, R. D., Monson, N., & Beatty, P. A. (1989). Affective judgments by patients with Parkinson’s disease or chronic progressive multiple sclerosis. *Bulletin of the Psychonomic Society*, *27*(4), 361-364.

Berneiser, J., Wendt, J., Grothe, M., Kessler, C., Hamm, A. O., & Dressel, A. (2014). Impaired recognition of emotional facial expressions in patients with multiple sclerosis. *Multiple sclerosis and related disorders*, *3*(4), 482-488.

Bisecco, A., Altieri, M., Santangelo, G., Di Nardo, F., Docimo, R., Caiazzo, G., ... & Gallo, A. (2020). Resting-state functional correlates of social cognition in multiple sclerosis: an explorative study. *Frontiers in behavioral neuroscience*, *13*, 276.

Bruno, D., Galiani, A., Golfeder, M., Pagani Cassará, F., Duncan, J., Sinay, V., & Roca, M. (2022). Perspective taking deficits and their relationship with theory of mind abilities in patients with relapsing-remitting multiple sclerosis (RRMS). *Applied Neuropsychology: Adult*, 1-11.

Cecchetto, C., Aiello, M., D’Amico, D., Cutuli, D., Cargnelutti, D., Eleopra, R., & Rumiati, R. I. (2014). Facial and bodily emotion recognition in multiple sclerosis: the role of alexithymia and other characteristics of the disease. *Journal of the International Neuropsychological Society*, *20*(10), 1004-1014.

Chanial, C., Basaglia-Pappas, S., Jacqueline, S., Boulange, A., Gourdon, C., Donya, S., ... & Borg, C. (2020). Assessment of implicit language and theory of mind in multiple sclerosis. *Annals of physical and rehabilitation medicine*, *63*(2), 111-115.

Czekóová, K., Shaw, D. J., Saxunová, K., Dufek, M., Mareček, R., Vaníček, J., & Brázdil, M. (2019). Impaired self-other distinction and subcortical gray-matter alterations characterize socio-cognitive disturbances in multiple sclerosis. *Frontiers in Neurology*, *10*, 525.

Dulau, C., Deloire, M., Diaz, H., Saubusse, A., Charre-Morin, J., Prouteau, A., & Brochet, B. (2017). Social cognition according to cognitive impairment in different clinical phenotypes of multiple sclerosis. *Journal of neurology*, *264*, 740-748.

Ehrlé, N., Espi, P., Labire, J., Loizeau, A., Menard, C., & Bakchine, S. (2020). Impairments of humour comprehension in multiple sclerosis. *Multiple sclerosis and related disorders*, *39*, 101443.

Fereydouni, S., Hadianfard, H., & Ashjazadeh, N. (2019). Facial emotion recognition in patients with relapsing-remitting multiple sclerosis. *Neurology Asia*, *24*(4).

Genova, H. M., Cagna, C. J., Chiaravalloti, N. D., DeLuca, J., & Lengenfelder, J. (2016). Dynamic assessment of social cognition in individuals with multiple sclerosis: a pilot study. *Journal of the International Neuropsychological Society*, *22*(1), 83-88.

Genova, H. M., & McDonald, S. (2020). Social cognition in individuals with progressive multiple sclerosis: a pilot study using TASIT-S. *Journal of the International Neuropsychological Society*, *26*(5), 539-544.

Gleichgerrcht, E., Tomashitis, B., & Sinay, V. (2015). The relationship between alexithymia, empathy and moral judgment in patients with multiple sclerosis. *European Journal of Neurology*, *22*(9), 1295-1303.

Goitia, B., Bruno, D., Abrevaya, S., Sedeño, L., Ibáñez, A., Manes, F., ... & Roca, M. (2020). The relationship between executive functions and fluid intelligence in multiple sclerosis. *PloS one*, *15*(4), e0231868.

Golde, S., Heine, J., Pöttgen, J., Mantwill, M., Lau, S., Wingenfeld, K., ... & Gold, S. M. (2020). Distinct functional connectivity signatures of impaired social cognition in multiple sclerosis. *Frontiers in Neurology*, *11*, 507.

Hälbig, T. D., Wüstenberg, T., Giess, R. M., Kunte, H., Bellmann‐Strobl, J., Ruprecht, K., & Paul, F. (2020). Emotional experience in patients with clinically isolated syndrome and early multiple sclerosis. *European Journal of Neurology*, *27*(8), 1537-1545.

Henry, J. D., Phillips, L. H., Beatty, W. W., McDonald, S., Longley, W. A., Joscelyne, A., & Rendell, P. G. (2009). Evidence for deficits in facial affect recognition and theory of mind in multiple sclerosis. *Journal of the International Neuropsychological Society*, *15*(2), 277-285.

Henry, A., Tourbah, A., Chaunu, M. P., Rumbach, L., Montreuil, M., & Bakchine, S. (2011). Social cognition impairments in relapsing-remitting multiple sclerosis. *Journal of the International Neuropsychological Society*, *17*(6), 1122-1131.

Henry, J. D., Von Hippel, W., Molenberghs, P., Lee, T., & Sachdev, P. S. (2016). Clinical assessment of social cognitive function in neurological disorders. *Nature Reviews Neurology*, *12*(1), 28-39.

Henry, A., Tourbah, A., Chaunu, M. P., Bakchine, S., & Montreuil, M. (2017). Social cognition abilities in patients with different multiple sclerosis subtypes. *Journal of the International Neuropsychological Society*, *23*(8), 653-664.

Henry, A., Lannoy, S., Chaunu, M. P., Tourbah, A., & Montreuil, M. (2022). Social cognition and executive functioning in multiple sclerosis: A cluster‐analytic approach. *Journal of neuropsychology*, *16*(1), 97-115.

Ignatova, V. G., Surchev, J. K., Stoyanova, T. G., Vassilev, P. M., Haralanov, L. H., & Todorova, L. P. (2020). Social cognition impairments in patients with multiple sclerosis: comparison with grade of disability. *Neurology India*, *68*(1), 94.

Isernia, S., Baglio, F., d’Arma, A., Groppo, E., Marchetti, A., & Massaro, D. (2019). Social mind and long-lasting disease: focus on affective and cognitive theory of mind in multiple sclerosis. *Frontiers in Psychology*, *10*, 218.

Isernia, S., Cabinio, M., Pirastru, A., Mendozzi, L., Di Dio, C., Marchetti, A., ... & Baglio, F. (2020). Theory of mind network in multiple sclerosis: a double disconnection mechanism. *Social neuroscience*, *15*(5), 544-557.

Jehna, M., Neuper, C., Petrovic, K., Wallner-Blazek, M., Schmidt, R., Fuchs, S., ... & Enzinger, C. (2010). An exploratory study on emotion recognition in patients with a clinically isolated syndrome and multiple sclerosis. *Clinical neurology and neurosurgery*, *112*(6), 482-484.

Jehna, M., Langkammer, C., Wallner-Blazek, M., Neuper, C., Loitfelder, M., Ropele, S., ... & Enzinger, C. (2011). Cognitively preserved MS patients demonstrate functional differences in processing neutral and emotional faces. *Brain imaging and behavior*, *5*, 241-251.

Koubiyr, I., Dulau, C., Deloire, M., Saubusse, A., Charre-Morin, J., Brochet, B., & Ruet, A. (2021, June). Structural and functional connectivity of the amygdala explain social cognition performances in multiple sclerosis. In *EUROPEAN JOURNAL OF NEUROLOGY* (Vol. 28, pp. 173-173). 111 RIVER ST, HOBOKEN 07030-5774, NJ USA: WILEY.

Kraemer, M., Herold, M., Uekermann, J., Kis, B., Wiltfang, J., Daum, I., ... & Abdel-Hamid, M. (2013). Theory of mind and empathy in patients at an early stage of relapsing remitting multiple sclerosis. *Clinical neurology and neurosurgery*, *115*(7), 1016-1022.

Kuzu Kumcu, M., Tezcan Aydemir, S., Ölmez, B., Durmaz Çelik, N., & Yücesan, C. (2022). Masked face recognition in patients with relapsing–remitting multiple sclerosis during the ongoing COVID-19 pandemic. *Neurological Sciences*, 1-8.

Labbe, T. P., Zurita, M., Montalba, C., Ciampi, E. L., Cruz, J. P., Vasquez, M., ... & Cárcamo, C. (2020). Social cognition in multiple sclerosis is associated to changes in brain connectivity: a resting-state fMRI study. *Multiple Sclerosis and Related Disorders*, *45*, 102333.

Labbe, T. P., Montalba, C., Zurita, M., Ciampi, E. L., Cruz, J. P., Vasquez, M., ... & Cárcamo, C. (2021). Regional brain atrophy is related to social cognition impairment in multiple sclerosis. *Arquivos de Neuro-Psiquiatria*, *79*, 666-675.

Lancaster, K., Stone, E. M., & Genova, H. M. (2019). Cognitive but not affective theory of mind deficits in progressive MS. *Journal of the International Neuropsychological Society*, *25*(8), 896-900.

Lenne, B., Barthelemy, R., Nandrino, J. L., Sequeira, H., Pinti, A., Mecheri, H., ... & Hautecoeur, P. (2014). Impaired recognition of facial emotional expressions in multiple sclerosis. *Neuropsychological Trends*, *15*(15), 67-83.

Massano, C., Lima, M., Monteiro, I., Machado, R., Correia, I., Nunes, C. C., ... & Batista, S. (2021). Outcomes on Social and Classic Cognition in adults with Pediatric-onset Multiple Sclerosis. *Multiple Sclerosis and Related Disorders*, *53*, 103071.

Mike, A., Strammer, E., Aradi, M., Orsi, G., Perlaki, G., Hajnal, A., ... & Illes, Z. (2013). Disconnection mechanism and regional cortical atrophy contribute to impaired processing of facial expressions and theory of mind in multiple sclerosis: a structural MRI study. *PLoS One*, *8*(12), e82422.

Montembeault, M., Brando, E., Charest, K., Tremblay, A., Roger, É., Duquette, P., & Rouleau, I. (2022). Multimodal emotion perception in young and elderly patients with multiple sclerosis. *Multiple Sclerosis and Related Disorders*, *58*, 103478.

Neuhaus, M., Bagutti, S., Yaldizli, Ö., Zwahlen, D., Schaub, S., Frey, B., ... & Penner, I. K. (2018). Characterization of social cognition impairment in multiple sclerosis. *European journal of neurology*, *25*(1), 90-96.

Ouellet, J., Scherzer, P. B., Rouleau, I., Métras, P., Bertrand-Gauvin, C., Djerroud, N., ... & Duquette, P. (2010). Assessment of social cognition in patients with multiple sclerosis. *Journal of the International Neuropsychological Society*, *16*(2), 287-296.

Pfaff, L., Gounot, D., Chanson, J. B., de Seze, J., & Blanc, F. (2021). Emotional experience is increased and emotion recognition decreased in multiple sclerosis. *Scientific Reports*, *11*(1), 21885.

Phillips, L. H., Henry, J. D., Scott, C., Summers, F., Whyte, M., & Cook, M. (2011). Specific impairments of emotion perception in multiple sclerosis. *Neuropsychology*, *25*(1), 131.

Pinto, C., Gomes, F., Moreira, I., Rosa, B., Santos, E., Silva, A. M., & Cavaco, S. (2012). Emotion recognition in multiple sclerosis.

Pitteri, M., Genova, H., Lengenfelder, J., DeLuca, J., Ziccardi, S., Rossi, V., & Calabrese, M. (2019). Social cognition deficits and the role of amygdala in relapsing remitting multiple sclerosis patients without cognitive impairment. *Multiple Sclerosis and Related Disorders*, *29*, 118-123.

Pöttgen, J., Dziobek, I., Reh, S., Heesen, C., & Gold, S. M. (2013). Impaired social cognition in multiple sclerosis. *Journal of Neurology, Neurosurgery & Psychiatry*, *84*(5), 523-528.

Prochnow, D., Donell, J., Schäfer, R., Jörgens, S., Hartung, H. P., Franz, M., & Seitz, R. J. (2011). Alexithymia and impaired facial affect recognition in multiple sclerosis. *Journal of neurology*, *258*, 1683-1688.

Radlak, B., Cooper, C., Summers, F., & Phillips, L. H. (2021). Multiple sclerosis, emotion perception and social functioning. *Journal of neuropsychology*, *15*(3), 500-515.

Raimo, S., Trojano, L., Pappacena, S., Alaia, R., Spitaleri, D., Grossi, D., & Santangelo, G. (2017). Neuropsychological correlates of theory of mind deficits in patients with multiple sclerosis. *Neuropsychology*, *31*(7), 811.

Realmuto, S., Dodich, A., Meli, R., Canessa, N., Ragonese, P., Salemi, G., & Cerami, C. (2019). Moral cognition and multiple sclerosis: a neuropsychological study. *Archives of Clinical Neuropsychology*, *34*(3), 319-326.

Roca, M., Manes, F., Gleichgerrcht, E., Ibáñez, A., De Toledo, M. E. G., Marenco, V., ... & Sinay, V. (2014). Cognitive but not affective theory of mind deficits in mild relapsing-remitting multiple sclerosis. *Cognitive and Behavioral Neurology*, *27*(1), 25-30.

Sofologi, M., Koutsouraki, E., Tsolaki, M., Tsolaki, A., Koukoulidis, T., Theofilidis, A., ... & Moraitou, D. (2019). Analyzing social cognition and understanding of social inferences in patients with multiple sclerosis. A comparative study. *Hellenic Journal of Nuclear Medicine*, *22*, 15-26.

Turner, J. A., Padgett, C., McDonald, S., Ahuja, K. D., Francis, H. M., Lim, C. K., & Honan, C. A. (2021). Innate immunity impacts social-cognitive functioning in people with multiple sclerosis and healthy individuals: Implications for IL-1ra and urinary immune markers. *Brain, Behavior, & Immunity-Health*, *14*, 100254.

Weinstein, A., Patterson, K. M., & Rao, S. (1996). Hemispheric asymmetries and processing of affective stimuli: Contribution of callosal communication. *Brain and Cognition*, *32*(2), 223-226.

Yap, S. M., Davenport, L., Cogley, C., Craddock, F., Kennedy, A., Gaughan, M., ... & McGuigan, C. (2023). Word finding, prosody and social cognition in multiple sclerosis. *Journal of Neuropsychology*, *17*(1), 32-62.

Yokote, H., Okano, K., & Toru, S. (2021). Theory of mind and its neuroanatomical correlates in people with multiple sclerosis. *Multiple Sclerosis and Related Disorders*, *55*, 103156.
